# Reliability and safety of anaesthetic equipment around an high-field 7-Tesla MRI scanner

**DOI:** 10.1016/j.bja.2023.02.019

**Published:** 2023-03-28

**Authors:** Philippa Bridgen, Shaihan Malik, Thomas Wilkinson, John N. Cronin, Tahzeeb Bhagat, Nicholas Hart, Stuart Mc Corkell, Joanne Perkins, Shane Tibby, Sara Hanna, Richard Kirwan, Thomas Pauly, Arthur Weeks, Geoff Charles-Edwards, Francesco Padormo, David Stell, Kariem El-Boghdadly, Sebastien Ourselin, Sharon L. Giles, Anthony D. Edwards, Joseph V. Hajnal, Benjamin J. Blaise

**Affiliations:** 1 Center for the Developing Brain, School of Biomedical Engineering and Imaging Sciences, King’s College London, St. Thomas’ Hospital, London, UK; 2 Biomedical Engineering Department, School of Biomedical Engineering and Imaging Sciences, London, UK; 3 London Collaborative Ultra High Field System (LoCUS), London, UK; 4 Department of Anaesthetics, St Thomas’ Hospital, London, UK; 5 Department of Paediatric Anaesthetics, London, UK; 6 Lane Fox Clinical Respiratory Physiology Research Centre, London, UK; 7 Lane Fox Respiratory Service, London, UK; 8 Centre for Human and Applied Physiological Sciences, King’s College London, London, UK; 9 Department of Paediatric Intensive Care, London, UK; 10 Drägerwerk AG & Co. KGaA, Lübeck, Germany; 11 Philips, MRPC, Orlando, FL, USA; 12 Department of Medical Physics, Guy’s and St Thomas’ NHS Foundation Trust, London, UK; 13 Department of Neonatology, Evelina London Children’s Hospital, Guy’s and St Thomas’ NHS Foundation Trust, London, UK

**Keywords:** anaesthesia machine, magnetic resonance imaging, safety, technology, ultra-high field MRI

Editor—Resolution achieved by ultra-high-field magnetic resonance imaging (MRI) allows precise anatomical and functional imaging studies.^1,2^ However, it relies on patients tolerating scans with lengthy acquisition time in a long narrow bore.^3^ General anaesthesia is routinely provided in MRI scanners operating at magnetic fields up to 3 Tesla with dedicated MR conditional equipment,^4–7^ however, this has not previously been tested in 7-Tesla (7T) MRI scanner environments. We investigated the reliability of an MR Conditional Fabius MRI Dräger anaesthetic machine (Dräger, Lübeck, Germany) and Philips Invivo MR400 monitoring system (Philips, Amsterdam, The Netherlands) around a 7T Siemens Magnetom Terra scanner (Siemens AG, Munich, Germany), as part of our safety and efficacy evaluation of anaesthesia delivery around a 7T MRI scanner (Fig 1).

A testing strategy was vetted by a steering committee (see supplementary file for steering committee composition and Supplementary Fig. S1 for testing strategy) and confirmed by our Hospital Clinical Governance Committee. We tested the ventilatory parameters and monitoring of patients using the appropriate standards (ISO 80601-2-12, 80601-2-13, 80601-2-61, and 80601-2-55 standards).

Configuration tests with the MR conditional anaesthetic machine outside the 40 mT line showed that 4.8-m-long breathing circuits (length required to reach the tracheal tube inside the bore of the magnet) and south-facing tracheal tubes would allow adequate patient positioning without artifacts. An IngMar ASL 5000 breathing simulator (IngMar Medical, Pitts-burgh, PA, USA) simulated four profiles (healthy neonate; toddler; child; and adult; Supplementary Table S1). Routine calibration and checks of the MR conditional anaesthetic machine were performed before tests.^8^ Comparisons were run between the anaesthetic machine placed in the anaesthetic bay (non-magnetic baseline) *vs* the 7T scanner room (the anaesthetic bay is 6 m away from the 0.5 mT line, with the MRI control room in between). All equipment was reviewed before and after testing by Trust Medical Physics and Medical Equipment Management Services. Following the aforementioned standards, tolerance ranges were defined by plus or minus 5 ml + 10% of the expected volume and plus or minus 3 cm H_2_O + 5% of the set pressure. MATLAB (MathWorks, Inc., Natick, MA, USA) routines were used to measure breath volumes (maximal; mean; and total volumes), pressures (plateau and positive end-expiratory pressures) and compare distribution means using Monte Carlo simulations (random sampling of 10 breaths repeated 1000 times).

Ventilation profiles were similar (Supplementary Figs S2–S5), and all measurements were within the tolerance ranges, even after long exposure to the static magnetic field. Effects of time-varying gradients were negligible (Supplementary Fig. S6). Coupling issues occurred between the breathing simulator and anaesthetic machine in pressure-support mode for the neonate model, although use of this ventilation mode is not standard practice in neonates. We also observed a more rapid decay in ventilation pressures during the expiratory phase in the pressure-controlled, volume-controlled, and spontaneously breathing modes in the non-magnetic environment, while the peak and plateau pressures were similar (Supplementary Figs S2–S4). Decays were variable from one mode to the other, but of small magnitude. We suggest the differences in expiratory decay are explained by a mechanical element, such as an interaction with a valve opening or closing mechanism that would be slower in the magnetic environment. The Hall effect might cause offsets within the flow sensors and it might also make mechanical components move slower. This could lead to an increase in minute ventilation which, nevertheless, would be within the stated tolerances.

The difference in expiratory decay could be missed when switching between the anaesthetic and examination rooms (in a clinical setting, we swap from a standard anaesthetic machine in the anaesthetic room to an MR conditional machine next to the MRI scanner, and breathing circuits are longer in the magnetic environment). Anaesthetists running MRI lists under general anaesthesia usually assess and adjust ventilation parameters once the patient is ventilated by the MR conditional anaesthetic machine. It is quite usual to use pressure levels on the MR conditional anaesthetic machine of a few cm H_2_O above the parameters used on the standard anaesthetic machine. This is part of MR conditional anaesthetic machine induction in our department.

For each monitoring component (wireless ECG, pulse oximetry), we performed measurements of the magnetically induced displacement force and torque based upon ASTM F2052-15 and F2213-17. A maximum deflection angle of 5 degrees was measured without torque, demonstrating minimal projectile/torque risk at 7T. Further radiofrequency heating tests did not demonstrate a significant increase in temperature (Supplementary Figs S7–S9).

After ethical and institutional approval, a test on an awake healthy adult volunteer was conducted. Baseline parameters were evaluated after a 10-min rest lying on an MR conditional trolley (using a Philips IntelliVue X2 monitor). We observed satisfactory comparative data between the baseline and observations acquired with the MR conditional Invivo MR400 expression monitoring in the 7T room. A Bland–Altman analysis was out of the scope of this study, but this analysis is expected to be part of future ultra-high field anaesthesia scans when more comparative data are available. Temperature and invasive blood pressure monitoring are also available with the Philips Invivo MR400 monitoring system. In the clinical setting, we usually measure patient temperature in the anaesthetic room before and after the scan for MR neuroimaging under general anaesthesia (average scan duration of 30 min). For MRI scans at 7T under general anaesthesia, we will use continuous temperature monitoring.

A 45-min brain scan was completed without complications, and monitoring performed adequately when challenged (simulation of apnoea by breath holding, hyperventilation, desaturation using a tourniquet on the arm, modification of inspired fraction in oxygen, administration of nitrous oxide, equipment or power supply disconnection). There were no heating reports where ECG leads had been positioned. Our entire epilepsy protocol was tested without the anaesthetic equipment, with the anaesthetic equipment off, and with the anaesthetic equipment switched on. Of clinical importance, there were no effects of the anaesthetic equipment on image quality (Supplementary Figs S10 and S11).

Following implementation of safety checklists, risk assessment, and simulation training, we described anaesthetic capacity at 7T ‘ultra-high-field anaesthesia’ which was approved as a new procedure. Anaesthetic risks were deemed equivalent to those near a 1.5T or 3T MRI scanner, ruling out the requirement of a human safety study. However, ongoing field testing and monitoring will be an essential part of clinical practice. We acknowledge that these recommendations should not be generalised to other MR conditional anaesthetic devices/7T MRI scanner pairs.

In conclusion, MR conditional anaesthetic equipment is reliable and safe to deliver anaesthesia in the 7T MRI environment. Institutions introducing 7T MR scanners will need to test their anaesthesia machines and monitoring equipment within the specific local environment, as conditions and field strengths vary between makes of scanners. These tests must involve the local MR safety expert who will be able to advise. Anaesthetists should be involved at all stages of planning to ensure that patient flow is safe and the monitoring conforms to national standards,^9^ which might require testing of equipment to develop a similar protocol to that used here.

## Supplementary Material

Supplementary data to this article can be found online at https://doi.org/10.1016/j.bja.2023.02.019.

SI file

## Figures and Tables

**Fig 1:**
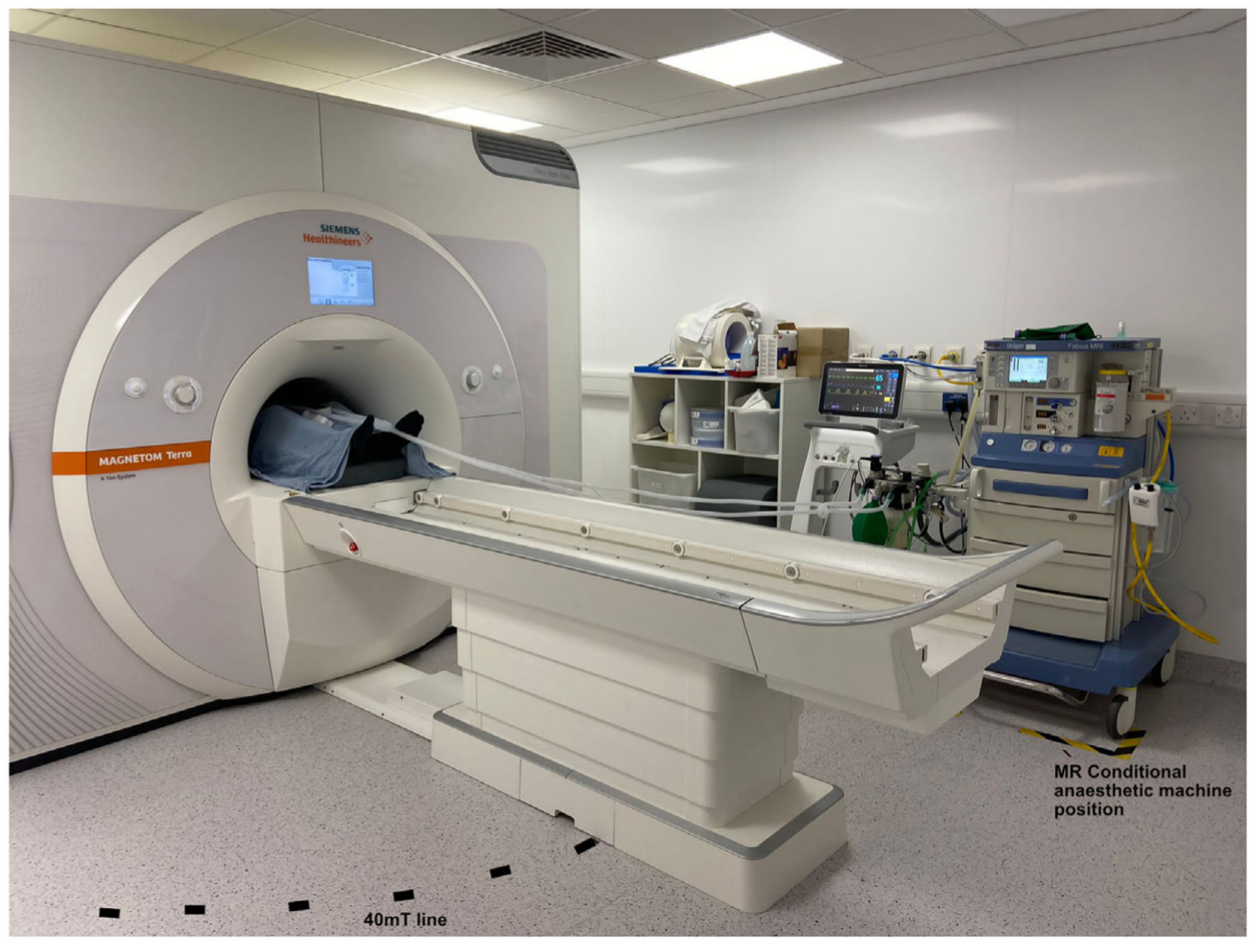
Anaesthetic set-up for ultra-high-field anaesthesia, including an MR conditional Fabius MRI Dräger anaesthesia machine (Dräger, Lübeck, Germany) and Philips Invivo MR400 monitoring system (Philips, Amsterdam, The Netherlands) around a 7T Siemens Magnetom Terra scanner (Siemens AG, Munich, Germany). The black and yellow dashed lines represent where the MR conditional anaesthesia machine should be positioned. The black dashed line around the MRI scanner represents the 40 mT line.
