# Supplementary material for: Reliability and safety of anaesthetic equipment around an high-field 7-Tesla MRI scanner
Source: Br J Anaesth. Author manuscript; Available in PMC 2024 Jul 11. (PMC7616168; doi:10.1016/j.bja.2023.02.019)
Supplement: SI file [file EMS197129-supplement-SI_file.docx]

**Reliability and safety of anaesthetic equipment around a 7T MRI scanner**

*Ultra-high Field Anaesthesia*

**Supplementary materials**

Philippa Bridgen,^1,2,3^ Shaihan Malik,^1,2,3^ Thomas Wilkinson,^1,2^ John N. Cronin,^4^ Tahzeeb Bhagat,^5^ Nicholas Hart,^6,7,8^ Stuart Mc Corkell,^4^ Joanne Perkins,^9^ Shane Tibby,^9^ Sara Hanna,^9^ Richard Kirwan,^4,5^ Thomas Pauly,^10^ Arthur Weeks,^11^ Geoff Charles-Edwards,^2,12^ Francesco Padormo,^12^ David Stell,^12^ Kariem El-Boghdadly,^4^ Sebastien Ourselin,^3^ Sharon L. Giles,^1,2^ Anthony D. Edwards,^1,2,13^ Joseph V. Hajnal^1,2^ and Benjamin J. Blaise^1,5^

1 Center for the Developing Brain, School of Biomedical Engineering and Imaging Sciences, King’s College London, St. Thomas’ Hospital, London, UK

2 Biomedical Engineering Department, School of Biomedical Engineering and Imaging Sciences, King's College London, London, UK

3 London Collaborative Ultra high field System (LoCUS), London, UK, Kings College London, London, United Kingdom

4 Department of Anaesthetics, St Thomas’ Hospital, Guy’s and St Thomas’ NHS Foundation Trust, London, UK

5 Department of Paediatric Anaesthetics, Evelina London Children’s Hospital, Guy’s and St Thomas’ NHS Foundation Trust, London, UK

6 Lane Fox Clinical Respiratory Physiology Research Centre, Guy's and St Thomas' NHS Foundation Trust, London, UK.

7 Lane Fox Respiratory Service, Guy's and St Thomas' NHS Foundation Trust, London, UK.

8 Centre for Human and Applied Physiological Sciences, King's College London, London, London, UK.

9 Department of Paediatric Intensive Care, Evelina London Children’s Hospital, Guy’s and St Thomas’ NHS Foundation Trust, London, UK

10 Drägerwerk AG & Co. KGaA, Moislinger Allee 53-55, D-23558 Lübeck, Germany

11 Philips, MRPC, Orlando, Florida, USA

12 Medical Physics, Guy's & St Thomas' NHS Foundation Trust, London, UK

13 Department of Neonatology, Evelina London Children’s Hospital, Guy’s and St Thomas’ NHS Foundation Trust, London, UK

Correspondence to: BJ Blaise

Email: Benjamin.blaise@nhs.net

Keywords: ultra-high field, magnetic resonance imaging, anaesthesia

**Acknowledgements**

This work was supported by a Wellcome Trust Collaboration in science award [WT201526/Z/16/Z], by core funding from the Wellcome/EPSRC Centre for Medical Engineering [WT203148/Z/16/Z] and by the National Institute for Health Research (NIHR) Biomedical Research Centre based at Guy’s and St Thomas’ NHS Foundation Trust and King’s College London and/or the NIHR Clinical Research Facility. The views expressed are those of the author(s) and not necessarily those of the NHS, the NIHR or the Department of Health and Social Care.

We also thank Dräger, Philips and Siemens for their support.

**Short title:**

**Ultra-high Field Anaesthesia**

***Steering committee composition:***

We selected an in-house steering committee covering all clinical, research, safety and institutional aspects impacted by the project (there were no independent members external to the Trust. Committee members work at St Thomas’ Hospital or the Evelina London Children’s Hospital, parts of Guy’s and St Thomas’ NHS Foundation Trust):

- Clinical Director for Children's Surgical Services and Paediatric Intensive Care
- Clinical Lead Paediatric Anaesthetics
- Clinical Governance Lead Paediatric Anaesthetics
- Research Lead Paediatric Anaesthetics
- Research Lead Paediatric Intensive Care
- Research and Development lead Evelina
- Research Lead Theatres, Anaesthesia and Perioperative medicine
- Clinical Governance Lead Theatres, Anaesthesia and Perioperative medicine
- Research Lead Intensive Care
- Clinical Director for Clinical Imaging and Medical Physics
- Director of Clinical & Research Imaging Operations
- Chair in Imaging Science
- Director Centre for the Developing Brain
- Magnetic Resonance safety lead
- Anaesthetic equipment lead
- Clinical engineering lead

**SI-figure 1.** Flowchart of the certification of ultra-high field anaesthesia at Guy’s and St Thomas’ NHS Foundation Trust. The human safety study was faded following the recommendation of the steering committee and decision of the clinical governance committee (ELCGG).

The steering committee evaluated all documents before submission to the Evelina London Clinical Governance Group (ELCGG), the Hospital Clinical Governance Board at the Evelina London Children’s Hospital. Incident reporting and review will be jointly evaluated by the steering committee, ELCGG and our Patient’s Safety Committee.

***Anaesthetic machine, monitoring and breathing equipment:***

The following equipment were used to carry out the tests, with MR conditionality limits specified in brackets:

- 7 T Siemens Magnetom Terra scanner.
- MR Conditional Fabius MRI Dräger anaesthetic machine (fringe field: 40mT).
- Nova Medical 1Tx32Rx head coil.
- MR Conditional Invivo MR400 Expression (fringe field: 500mT, time-weighted average RF magnetic field exposure: 7.2 microT, specific absorption rate: 4 W.Kg^-1^, magnetic field: 3 T). Associated emitting boxes being able to go through the scanner bore.
- IngMar ASL 5,000 artificial lung (non-MR Conditional equipment maintained outside the 5gauss line).
- LIR T360 infrared (IR) thermal camera.

***Patient profile simulation for breathing tests:***

| Case | 1 | 2 | 3 | 4 |
| --- | --- | --- | --- | --- |
| Weight (kg) | 3.5 | 7 | 20 | 75 |
| Expected Volume (ml) | 30 | 60 | 150 | 500 |
| Exp Frequency (min^-1^) | 35 | 25 | 20 | 12 |
| Peep (cmH_2_O) | 4 | 4 | 5 | 6 |
| FiO2 (%) | 35 | 35 | 35 | 35 |
| Resistance (cmH_2_O.l^-1^.s^-1^) | 20 | 20 | 20 | 6 |
| Compliance (ml.cmH_2_O^-1^) | 8 | 8 | 12 | 50 |
| Effort (cmH_2_O) | 4 | 5 | 8 | 11 |

**SI-table.** Simulated profiles with the artificial lung.

***Results on the anaesthetic machine***

*Uncertainties on the measurements and significance testing*

For each breath simulated by the artificial lung, we measured volume (maximal; mean; and total volumes per breath) and pressure (plateau and positive end-expiratory pressures per breath) parameters. Monte Carlo simulations were used to evaluate the means of each parameter of interest (random sampling of 10 breaths repeated 1000 times). Means of the distributions were compared and a t-test was used to assess if the difference could be considered as statistically significant.

Measurement uncertainties were evaluated for volumes and pressures. We calculated the difference between the maximum and minimum values of each measurement and divided it by 2. The average over all conditions was used to define the uncertainty. For volumes, the measurement uncertainties were 6ml and 1cmH_2_O for pressures for cases 1 to 3. Interestingly, similar coupling issues arose with pressure support ventilation, with measurement uncertainties dropping to 3ml and 1cmH_2_O when these ventilation modes are excluded from the calculation. This represents 6 % for Case 1, 4% for Case 2, 4% for Case 3 for volumes. Case 4 was treated separately due to the important difference in terms of volumes and pressures expected. Measurement uncertainties were 25ml and 1cmH_2_O for pressures. This represents 6% for Case 4 in terms of volumes.

All the differences reported between the external and magnetic environments were found to be statistically significant (p<0.01, including multiple hypothesis testing correction) using a paired t-test, however the size of differences was very small and within the stated tolerances.

*Spontaneous breathing*

Spontaneous breathing was simulated by increasing the muscular inspiratory pressure to levels leading to the expected volumes for the different cases. Anaesthetic machine was set up with the Man Spont mode. Volume variations were within the tolerance ranges defined above, respectively 1%, 3%, 8% and 6% for cases 1 to 4. Similar variations within the tolerance ranges were observed for inspiratory pressures and positive end-expiratory pressures, respectively 11%, 8%, 7% and 18% for the firsts; 8%, 6%, 11% and 13% for the seconds. These variations didn’t trigger any ventilation warnings or alarms during the simulation.

In all scenarios, the anaesthetic machine behaved as expected. It provided reliable and stable ventilation in the anaesthetic bay and in the magnetic environment, with really limited volume or pressure variations.

**SI-figure 2.** Volume curves for spontaneously breathing case 4 scenario, blue in the anaesthetic bay and red in the magnetic environment. Maxima are identified by green stars and minima by black diamonds.

*Pressure-controlled*

In pressure-controlled ventilation tests, muscular inspiratory pressures were inhibited. 2 levels of pressure were simulated for different scenarios (case 1: +10 and +15 cmH_2_O, case 3: +15 and +25 cmH_2_O, case 4: +15 and +25cmH_2_O). Scenario 2 wasn’t explored due to time constraints. Differences between ventilations in the anaesthetic bay and magnetic environment were:

- Case 1: 3% and 1% for volumes, 2% and 1% for inspiratory pressures, 2% and 1% for PEEP respectively.
- Case 3: 0% and 2% for volumes, 0% and 0% for inspiratory pressures, 1% and 1% for PEEP respectively.
- Case 4: 6% and 4% for volumes, 10% and 6% for inspiratory pressures, 7% and 4% for PEEP respectively.

Pressure-controlled mode provided steady ventilation in the anaesthetic bay and magnetic environment, with small variations not triggering any warnings or alarms during the simulation. Pressure-controlled mode was not tested with Case 2 due to time constraints.

**SI-figure 3.** Pressure curves for pressure-controlled case 1 scenario (+10/+4 cmH_2_O), blue in the anaesthetic bay and red in the magnetic environment. Plateau pressure is identified by black diamonds.

*Volume-controlled*

In volume-controlled ventilation tests, muscular inspiratory pressures were inhibited as well. Two levels of frequencies were simulated for different scenarios (case 1: 30 and 35min^-1^, case 2: 25 and 30min^-1^, case 3: 15 and 20min^-1^, case 4: 10 and 15min^-1^). Differences between ventilations in the anaesthetic bay and magnetic environment were:

- Case 1: 8% and 13% for volumes, 8% and 6% for inspiratory pressures, 8% and 10% for PEEP respectively.
- Case 2: 3% and 9% for volumes, 2% and 11% for inspiratory pressures, 1% and 4% for PEEP respectively.
- Case 3: 2% and 4% for volumes, 0% and 3% for inspiratory pressures, 0% and 3% for PEEP respectively.
- Case 4: 2% and 5% for volumes, 8% and 6% for inspiratory pressures, 7% and 9% for PEEP respectively.

**SI-figure 4.** Volume (top) and pressure (bottom) curves for volume-controlled in a healthy 20-kg child (15 breaths.min^-1^) in the anaesthetic bay (blue and green lines) and in the magnetic environment at ultra-high field (red and orange dotted lines).

Volume-controlled mode provided steady ventilation in the anaesthetic bay and magnetic environment, especially in cases 3 and 4. The small variations did not trigger any warnings or alarms during the simulation. Figure is presented in the main manuscript.

*Pressure support*

Pressure support was simulated by selecting an appropriate breathing effort (muscular inspiratory pressure of 5 cmH_2_O) capable of triggering ventilation support by the anaesthetic machine, with a trigger set up at 2l.min^-1^. Different levels of support were simulated scenarios (case 1: +15 cmH_2_O, case 2: +5 and +10 cmH_2_O, case 3: +5 and +10 cmH_2_O, case 4: +5 and +10 cmH_2_O). Differences between ventilations in the anaesthetic bay and magnetic environment were:

- Case 1: 7% for volumes, 7% for inspiratory pressures, 12% for PEEP respectively.
- Case 2: 6% and 2% for volumes, 3% and 9% for inspiratory pressures, 0% and 1% for PEEP respectively.
- Case 3: 4% and 2% for volumes, 3% and 13% for inspiratory pressures, 2% and 34% for PEEP respectively.
- Case 4: 8% and 7% for volumes, 2% and 0% for inspiratory pressures, 1% and 1% for PEEP respectively.

**SI-figure 5.** Volume curves for pressure-support case 2 scenario (inspiratory pressure +10 cmH_2_O). Blue in the anaesthetic bay and red in the magnetic environment.

*Effect of scanning*

MRI pulse sequences with strong field gradients were acquired to evaluate a potential effect on the anaesthetic machine. These were done on a case 3 volume-controlled simulation. Sequences with moderate (1) and strong (2) field gradients were used. The following differences were observed:

- Between the anaesthetic bay and magnetic environment with moderate gradient sequence: 3% for volumes, 3% for inspiratory pressures and 3% for PEEP.
- Between the anaesthetic bay and magnetic environment with strong gradient sequence: 3 % for volumes, 3% for inspiratory pressures and 3% for PEEP.
- In the magnetic environment with moderate gradient sequence compared to no scanning: 1% for volumes, 0% for inspiratory pressures and 0% for PEEP.
- In the magnetic environment with strong gradient sequence compared to no scanning: 2% for volumes, 0% for inspiratory pressures and 0% for PEEP.
- In the magnetic environment with moderate gradient sequence compared to strong gradient sequence: 0 % for volumes, 0% for inspiratory pressures and 0% for PEEP.

**SI-figure 6.** Volume curves for volume-controlled case 3 scenario frequency = 20min^-1^), top green in the magnetic environment no scanning, middle blue in the magnetic environment scanning with a sequence using moderate field gradients and bottom red in the magnetic environment scanning with a sequence using strong field gradients.

*Effect of long and repetitive exposure to magnetic field*

The anaesthetic machine was left for a month in the magnetic environment, with standard scanning on-going. Effect of long and repetitive exposure to magnetic field was specifically tested on a case 4 volume-controlled scenario (frequency 15min^-1^). The following differences were observed:

- In the anaesthetic bay, 1^st^ test versus 2^nd^ test: 7% for volumes, 6% for inspiratory pressures and 6% for PEEP.
- In the magnetic environment, 1^st^ test versus 2^nd^ test: 6% for volumes, 5% for inspiratory pressures and 16% for PEEP.

Differences were also small between the anaesthetic bay and magnetic environment for the 1^st^ test (volumes 2%, inspiratory pressures 4% and PEEP 2%) or the second (volumes 5%, inspiratory pressures 6% and PEEP 9%), as well as anaesthetic bay 1^st^ test and magnetic environment 2^nd^ test (volumes 2%, inspiratory pressures 4% and PEEP 14%) or magnetic environment 1^st^ test and anaesthetic bay 2^nd^ test (volumes 8%, inspiratory pressures 7% and PEEP 8%).

***Results on the monitoring equipment***

Evaluation of monitoring equipment was carried out in 3 phases: static magnetic field torque/attraction tests, RF heating tests, and a human test (healthy consenting adult).

*Static Magnetic Field Attraction/Torque Tests*

ECG and SpO_2_ emitting boxes were progressively moved towards the magnet isocentre to monitor magnetic attractions or torque forces. A pendulum test was also done with devices suspended on a 50cm long thread from a pivot point mounted on a protractor. Angles of deflection were noted. No perceivable magnetic forces were felt on ECG and SPO_2_ emitting boxes anywhere within the bore. There were no measurable deflection angles produced (<5 degrees).

*Radiofrequency Heating Tests*

A skin-on pork leg joint was used as a meat phantom to simulate human tissue. Usually emitting boxes, SpO_2_ probe and ECG leads would be outside the coil during anaesthesia, experiments were performed with the monitoring equipment placed in and in the vicinity of the Nova coil in multiple configurations designed to provoke a worst-case heating response (SI – figure 6). Scans were run for 12 minutes at maximum RF power (displayed SAR value=100% in first level mode) and temperatures were measured using a thermal infrared camera (FLIR T360). Since the camera could not see the coil when inside the scanner, the bed was withdrawn quickly after each scan test using the emergency table release (taking approximately 5s) with IR measurements made immediately after.


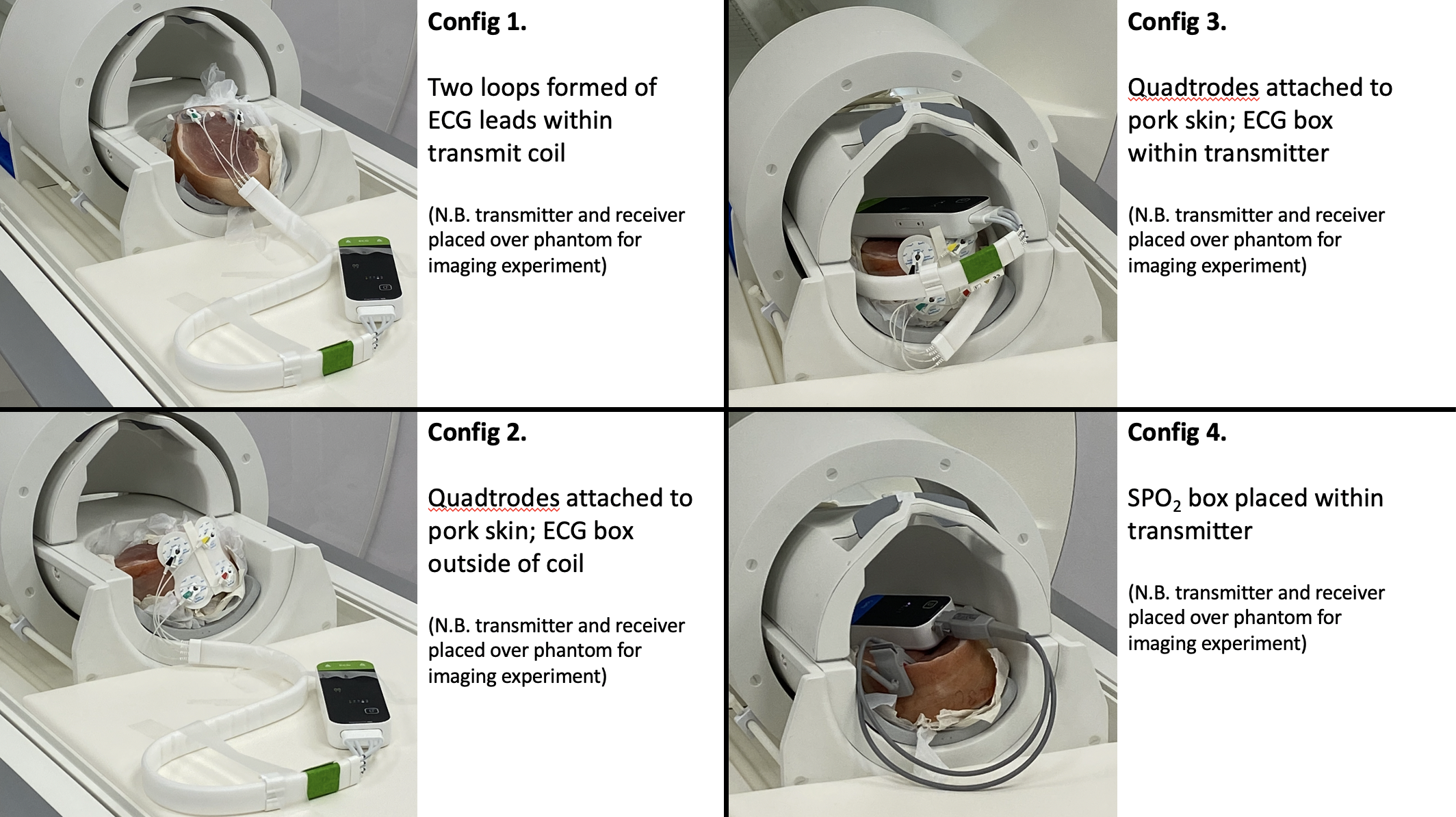


**SI-figure 7.** Experimental configurations used during radiofrequency heating tests. Each configuration was designed to provoke a worst-case heating scenario (looped ECG cables, transmit/receiver boxes placed within the RF fields of the coil).

We did not observe any significant increase in temperature in any of the configurations. Results are presented in SI - figures 7 and 8. Considering that the monitoring equipment will be further away from the coil during a UFA scan, it is unlikely that the Philips Expression MR400 monitoring devices would induce a heating risk when used at 7T.

***
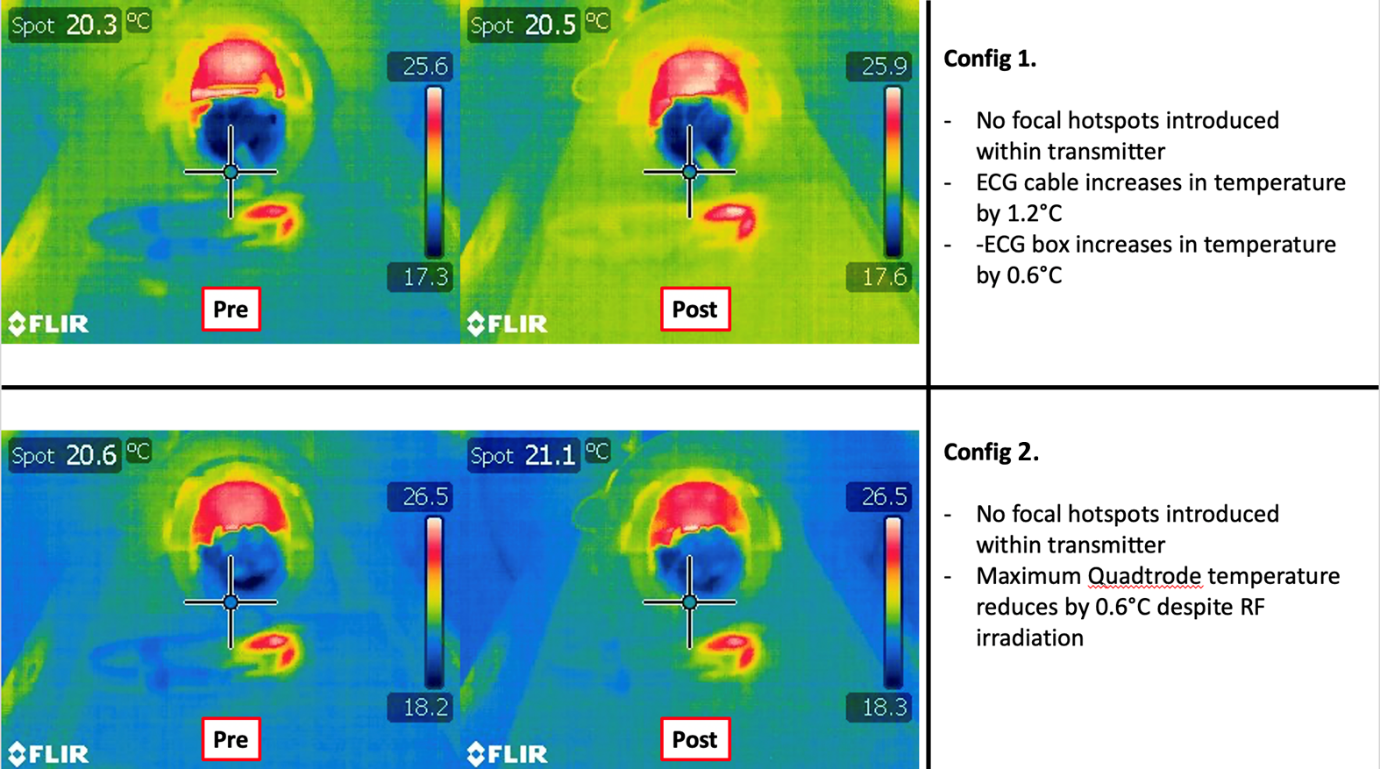
***

**SI-figure 8.** Temperature changes observed during radiofrequency heating tests for ECG leads and emitting box (configuration 1 and 2).


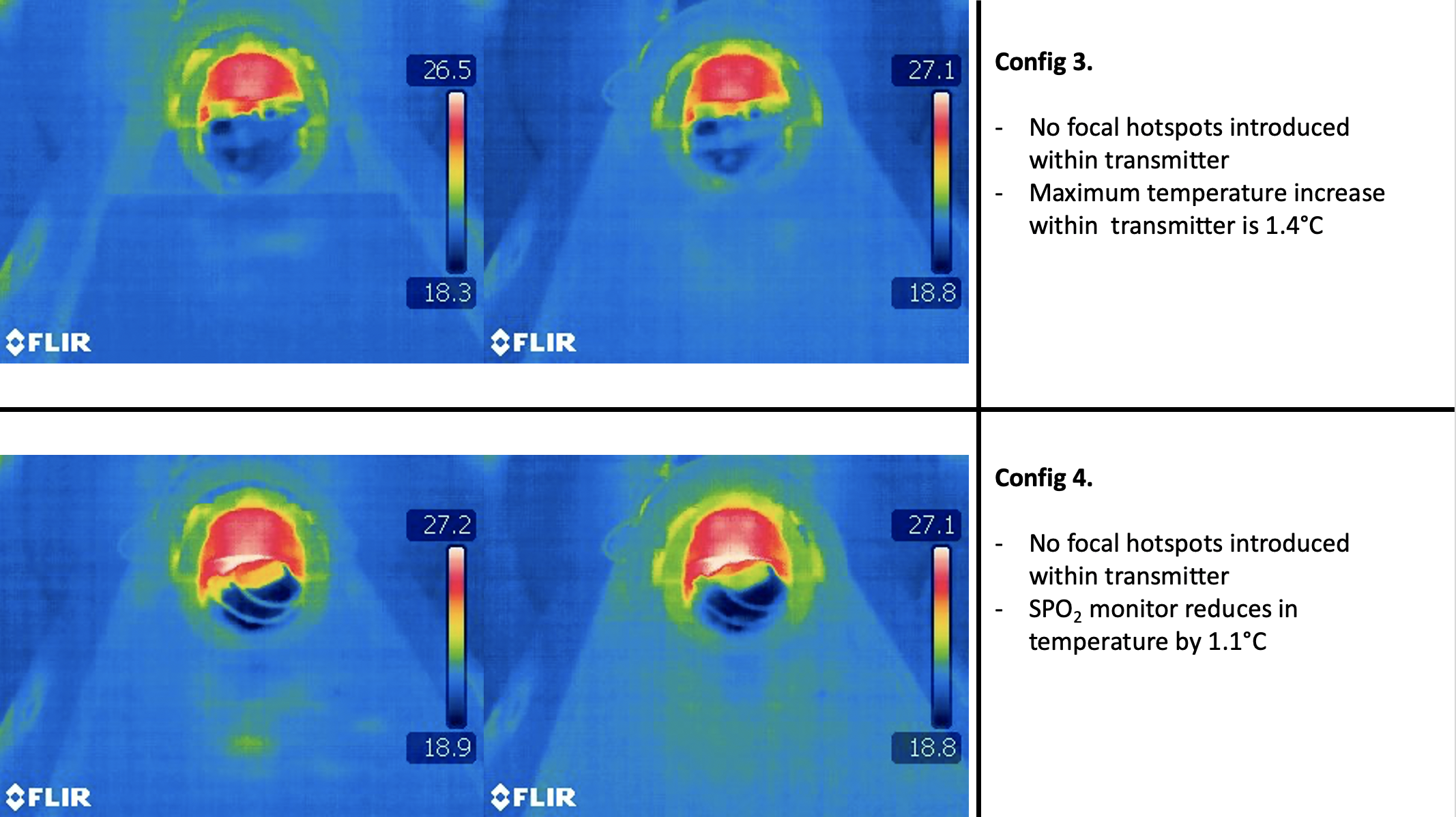


**SI-figure 9.** Temperature changes observed during radiofrequency heating tests for SpO_2_ probe and emitting box (configuration 3 and 4).

*Human test*

Following these tests, and based on ethical and regulatory approval, a healthy adult from our research centre volunteered to test the Invivo MR400 expression. Volunteer was awake throughout the experiment. Baseline parameters were evaluated after a 10min rest lying on a MR Conditional trolley using standard non-MR Conditional monitoring (Philips IntelliVue X2 monitoring with gas detection). MR Conditional saturation probe, ECG leads and their transmitters, non-invasive blood pressure cuff and temperature probe were safely positioned to limit any risks of RF induced burns.

Patient was transferred on the MR Conditional trolley to the scanning bed in the scanner room. Similar observations were measured, on the scanning bed outside the scanner, after transfer of the healthy volunteer to the magnet core and finally whilst scanning.

We observed a good match between the baseline observations (non-MR Conditional Philips IntelliVue X2) and those acquired in the 7T room (MR Conditional Invivo MR400 expression).

We also tested the validity of all the alarms, administration of different gases (oxygen and nitrous oxide), power disconnection. The healthy volunteer was monitored during a 45min head scan without any clinical or technical difficulties. Administration of nitrous oxide or increase in oxygen concentration was immediately picked up by the Invivo MR400 expression equipment. Power supply disconnection was uneventful with an alarm indicating the disconnection, the immediate engagement of battery power supply and the autonomy time left appearing on the screen.

We evaluated desaturation (tourniquet), apnea (breath holds), equipment disconnection alarms. Monitoring equipment reacted immediately providing adequate alarms. Voluntary fluctuations of respiratory rate by the healthy volunteer were immediately identified by the monitoring equipment. We failed to simulate arrhythmias, especially ventricular fibrillation or asystole (by rubbing the chest or misplacements of electrodes) due to the confined space and physical constraints inside the magnet core.

There were no reports of any heating or painful sensation by the healthy volunteer, a simple skin check didn’t identify any marks. The monitoring equipment operated as expected, whether on the MRI scanner bed or inside the core before, during and after scanning.

***Effect of the anaesthetic machine and monitoring equipment on the image quality***

We measured the effects of the presence of an MR Conditional anaesthetic machine and monitoring equipment on image quality by measuring noise spectra using the vendor’s standard noise spectrum scan with a phantom loaded into the RF coil.


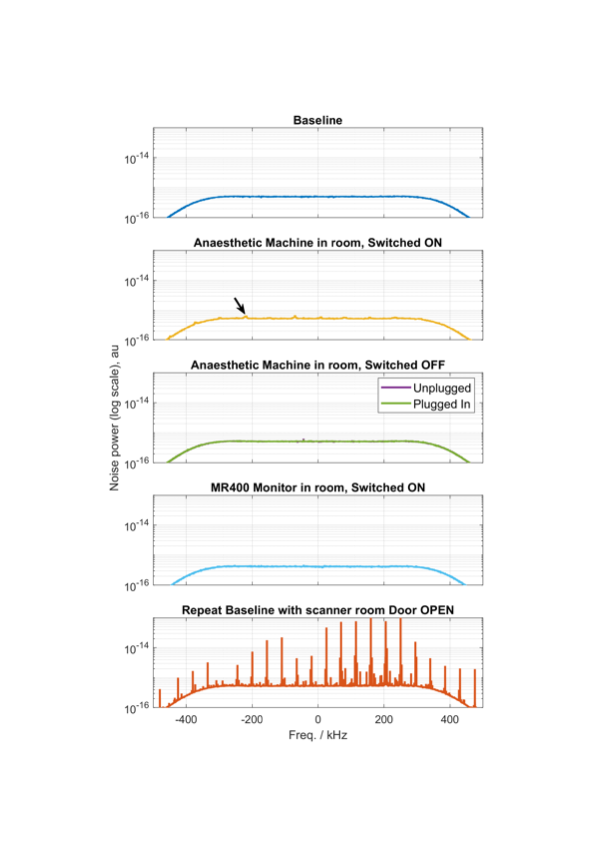


**SI-figure 10.** Noise power acquisition, top: baseline scan (no additional equipment in scanner room); second row: anaesthetic machine placed in the scanner room and switched on; third row: anaesthetic machine in scanner room but switched off, either plugged or unplugged from mains power; fouth row: monitoring equipment placed in the scanner room and switched on; fifth row: repeat of baseline (no anaesthetics or monitoring equipment) but with scanner room door open.

Noise power spectra were recorded without the additional equipment in the room (SI – figure 10 top panel, blue trace). With the MR Conditional anaesthetic machine in the room and powered on (second plot, yellow curve) very small changes of the noise spectrum could be seen (e.g. see arrow) but these are not significant enough to cause any measurable change in image quality. When the anaesthetic machine is powered off these small disturbances disappear and are not altered if the machine is either plugged in or not to the mains. The monitoring equipment didn’t change the background noise (fourth panel, light blue) even when powered on. As a reference we also measured noise power spectra in the baseline condition, but with the door to the RF screened room open – which is known to compromise image quality. It is clear from the fifth panel (orange trace) that in this case a significant amount of noise is detected, as the spikes on spectrum reach over two orders of magnitude higher than those from the anaesthetics equipment. Scanning with the screened room door open is known to introduce image artefacts - to reiterate this is not a required step for the use of the anaesthetic equipment, it is provided as a reference to give context to the almost non-existent effect seen from that equipment.

We acquired our whole epilepsy protocol (localiser, B1 map, GRE map, axial T2, FLAIR 3D, MP2RAGE, coronal oblique T2, sagittal T2, QSM, FLAIR 2D) on a healthy adult volunteer awake, without the anaesthetic equipment, with the anaesthetic equipment off and then switched on. An example is given in SI – figure 11, with the image on the left being recorded with the anaesthetic equipment off and the one on the right when the equipment is switched on. No differences can be seen on the image quality throughout the protocol.


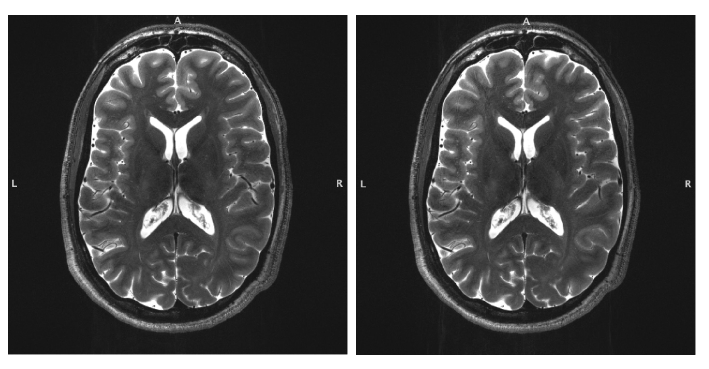


**SI-figure 11.** Axial T2 acquired at 7T with the anaesthetic machine and monitoring equipment off (left) and switched on (right).

***Human safety study***

Initial discussion on the test plan included the possibility of a human safety study following the initial non-clinical tests (SI – figure 1). Looking for single adverse events would require thousands of participants. Another way to look at the problem would be to consider the extension of certification of medical devices (post-approval studies). They usually require hundreds of participants. The sample size should be big enough to give confidence that the absence of adverse events means that the risks associated with the use of the MR compatible Fabius MRI Draeger anaesthetic machine and Invivo MR400 Expression monitoring at 7T were low enough to be outweighed by the likely clinical benefit.

Our hypothesis was that ultra-high field anaesthesia would be uneventful and this has been reinforced by our tests. The steering committee established that based on our tests, planning and management plan, the clinical risks would be similar to the risks of any MRI scans under general anaesthesia at lower fields within the Trust. A safety study was ruled out, as it is unlikely to bring any critical events that would differ from our usual practice at lower clinical fields, whilst delaying the access to ultra-high fields for our young and vulnerable patients. It was thus recommended to approve ultra-high field anaesthesia as a new procedure with the requirement to include a structured audit reporting system within our monitoring. This was acted by our Hospital Clinical Governance Committee, the Clinical Directors of the Directorates involved in the project, the Medical Director and the Patient’s Safety Committee.

Our monitoring phase will also include a structured audit report system focusing on technical and clinical issues. This should take the form of a prospective case-control study (matched controls being taken on our clinical MRI scanners operating at lower fields and using similar anaesthetic approach, ventilation strategy and monitoring).

In conclusion, our tests show that an MR Conditional Fabius MRI Dräger anaesthetic machine (Dräger, Lübeck, Germany) and Philips Invivo MR400 monitoring system (Philips, Amsterdam, Netherlands) can safely operate near a 7-Tesla (7T) Siemens Magnetom Terra scanner (Siemens AG, Munich, Germany), paving the way towards ultra-high field anaesthesia.
